# Beyond Corticoresistance, A Paradoxical Corticosensitivity Induced by Corticosteroid Therapy in Pediatric Acute Lymphoblastic Leukemias

**DOI:** 10.3390/cancers15102812

**Published:** 2023-05-18

**Authors:** Laure Angot, Pascale Schneider, Jean-Pierre Vannier, Souleymane Abdoul-Azize

**Affiliations:** 1 Normandie University, UNIROUEN, IRIB, Inserm, U1234, 76183 Rouen, France; 2 Department of Pediatric Immuno-Hemato-Oncology, Rouen University Hospital, 76038 Rouen, France

**Keywords:** acute lymphoblastic leukemia, glucocorticoid, resistance, signaling pathways

## Abstract

**Simple Summary:**

Although remarkable progress in the treatment of acute lymphoblastic leukemia (ALL) has been observed, some patients (~20%) relapse. Resistance to therapies is a hallmark of relapses and treatment failure in ALL. Such resistances may involve different cellular mechanisms, including the modulation by therapeutic drugs of cell survival signaling pathways that may lead to therapy-induced resistance. This article will begin by providing an update of mechanisms of resistance that may lead to therapy-induced resistance in ALL. It also provides proof of concept for the therapeutic exploitation of these signaling pathways to improve treatments.

**Abstract:**

Known as a key effector in relapse of acute lymphoblastic leukemia (ALL), resistance to drug-induced apoptosis, is tightly considered one of the main prognostic factors for the disease. ALL cells are constantly developing cellular strategies to survive and resist therapeutic drugs. Glucocorticoids (GCs) are one of the most important agents used in the treatment of ALL due to their ability to induce cell death. The mechanisms of GC resistance of ALL cells are largely unknown and intense research is currently focused on this topic. Such resistance can involve different cellular and molecular mechanisms, including the modulation of signaling pathways involved in the regulation of proliferation, apoptosis, autophagy, metabolism, epigenetic modifications and tumor suppressors. Recently, several studies point to the paradoxical role of GCs in many survival processes that may lead to therapy-induced resistance in ALL cells, which we called “paradoxical corticosensitivity”. In this review, we aim to summarize all findings on cell survival pathways paradoxically activated by GCs with an emphasis on previous and current knowledge on gene expression and signaling pathways.

## 1. Introduction

Acute lymphoblastic leukemias (ALL) are diseases resulting from transformations and mutations of hematopoietic cells. These mutations occur during cell development driving aberrant proliferation and survival of B and T cells [1]. Pediatric ALL developed in children until the age of 12 and present a peak prevalence between 2 and 5 years old [2]. The exact causes remain unclear, but some genetic, immunologic, viral and environmental factors seem to be implicated. Less than 5% of cases have been associated with inherited predisposing genetic syndromes such as Down’s syndrome, Bloom’s syndrome, ataxia-telangiectasia or Nijmegen breakage syndrome or with ionizing radiation [3,4]. ALL is the most common childhood malignancy with 80% of childhood leukemias and 25% of all childhood cancers [3]. Huge strides have been made over the past 50 years in the understanding of management of childhood ALL resulting in improvement in cure rates from approximately 10% to approximately 90% [3,5,6,7]. It has been shown that 85% of the cases of childhood ALL are of the B–lineage [3,8]. At the same time, T–ALL accounts for 10%–15% of the cases of childhood ALL and the outcome is more severe [9,10]. 

## 2. Glucocorticoids in Leukemia Treatment

Glucocorticoids (GCs) are small compounds derived from cortisol which is produced by the adrenal gland. Upon binding with GC, the glucocorticoid receptor, GR, encoded by the NR3C1 gene, dimerizes and migrates to the nucleus where it functions as a transcription factor and regulates the expression of multiple steroid-response genes [11,12]. GCs are involved in many biological processes including metabolism, development, differentiation and neural activity [13]. Because of their plural activity capacity, they became used as therapeutic agents in the treatment of many diseases. For example, GCs are used as anti-inflammatory or anti-proliferative treatments in immunopathies or leukemias, respectively. [13]. Nowadays, ALL patient care is based on chemotherapy protocols, in which GC take an important place (Table 1 and Table 2) [14]. At the beginning, prednisone was used in treatment. Protocols consisted of 4 weeks of prednisone in association with other drugs [14]. In recent years, dexamethasone, a synthetic analog of cortisol, which differs molecularly from prednisone by several atoms, has been increasingly used to treat ALL for its superior efficacy (for review see ref. [14]). For example, dexamethasone has an additional fluorine atom and an additional methyl group. These changes slow the metabolism of dexamethasone and lead to an extension of plasma half-life (200 min vs. 60 min for prednisolone) and biological half-life (36–54 h vs. 24–36 h) [14]. Nevertheless, while dexamethasone has increased efficiency, its cytotoxic effects are much more important than those of prednisone [14] and they should be considered.

Furthermore, the essential role of endogenous GC in normal cell physiology has been demonstrated, in particular via the interaction of the GR with the T cell receptor (TCR) to modulate T cell development [23,24] and homeostasis [25]. 

## 3. Glucocorticoids and Leukemic Cell Death: Mechanisms

GCs have a cytotoxic effect by binding to GR in the cytoplasm. Binding of GC to the GR triggers the dissociation of proteins bound to the receptor such as hsp and BAG-1 [26]. This activates the nuclear localization signal (NLS) domains of the receptor. After that, receptors are dimerized and translocate to the nucleus where they can interact with glucocorticoid-response elements, GRE. This interaction leads to the activation, inactivation or modulation of responsive genes and repress mostly the transcriptional activation of the activating protein-1 (AP-1), the nuclear factor (NF-kB) and even the GR itself [13]. Receptors can also stay in monomeric form and repress directly, by interaction, the activity of transcription factors AP-1 and NF-kB. The inhibition of these pathways induced cell-cycle arrest and apoptosis [13,14,27]. The pro-apoptotic response following GC binding to their receptors depends on their capacity to induce transcription of BCL2L11 which encodes the pro-apoptotic BH3-only factor BIM [28].

Two major apoptosis pathways can be distinguished: an intrinsic or mitochondrial pathway characterized by disruption of membrane potential, release of cytochrome c and activation of caspases [29] and pathways induced by membrane death receptors, such as TNFR1 or Fas, which bind their ligand (TNF-α, FasL etc.) and recruit a series of downstream factors, such as caspase 8, which leads to cell death. Importantly, these two pathways can be activated or modulated by GC [29]. In addition, GC might induce apoptosis indirectly by gene deregulation, which drives distress and cellular damage, such as production of oxygen radicals, alteration of metabolic pathways and Ca^2+^ fluxes [29]. However, many key components that associate Ca^2+^ signaling in GC sensitivity are not fully understood, as discussed below.

## 4. Glucocorticoids Stimulate Leukemic Cell Resistance: The Paradox

Despite its strong anti-inflammatory capacity, GC therapy is limited by two major drawbacks. First, GCs are well-known to be associated with side effects, particularly when it is administrated in high doses for long time periods. The toxic effects of the major GCs (prednisone and dexamethasone) are still investigated in ALL patients but are most often reported with dexamethasone causing sides effects such as myopathy, obesity or bone fracture [14]. 

In the meantime, some patients are refractory to the therapy and become GC-resistant. Yet, the mechanistic basis of GC resistance remain elusive [30]. Resistance can either be inherited, mostly via mutations [30] in the GR, NR3C1 gene [31] or other loss-of-functions mutations in the GR [32,33]. Resistance can also be induced after relapse; it has been shown that relapsed leukemia cells are more resistant to GC [34]. In addition to this secondary resistance observed during relapses, treatment of ALL by GCs is also limited by primary resistance, i.e., from the upfront GC treatment [35,36], and this initial response to GC therapy is a major predictor of response to chemotherapy and long-term B-ALL patient outcomes.

**Figure 1:**
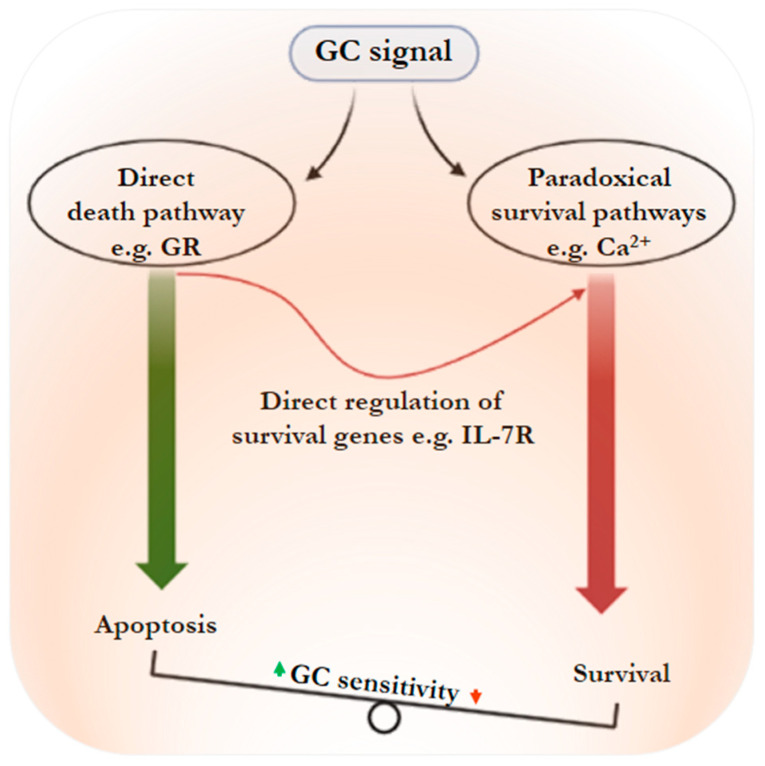
Proposed model for ALL cell fate following GC treatment. In this model, GC, by directly regulating crucial death or survival genes, leads to apoptotic cell death, but at the same time, paradoxically promotes ALL cell survival via several pathways (for other details see text).

Theoretically, resistance to GC treatment is manifested by the absence of a cell response (i.e., no cell death) or by genetic changes involved in the induction of cell death. We believe that this view is incomplete; indeed, the absence of cell death in response to GC treatment does not mean that the cells do not respond to GC. Here, we rather propose a “paradoxical corticosensitivity” because it turns out that, in addition to the absence of response to GC treatment, this treatment also activates cell survival pathways, contrary to the expected effect. Thus, this underlines a paradox in which GCs stimulate leukemic cell growth and resistance, rather than induce cell death. The activation of one or several pathways could be the cause of GC resistance in ALL. These pathways can act directly on the GR function, or indirectly by interfering with the GC-induced apoptosis signaling (Figure 1). A few pathways that play a role in GC-stimulated tumor ALL cell survival (Table 3) have been highlighted.

### 4.1. Calcium Signaling

Ca^2+^ is a versatile secondary messenger that regulates many cellular functions (cell proliferation, invasiveness, angiogenesis, migration and metastasis) by activating or inhibiting a variety of signaling pathways through Ca^2+^-dependent proteins [53,54,55]. Several reports have shown transient increases in intracellular Ca^2+^ signaling in multiple models after dexamethasone administration [37,38,56,57], but many key elements of that association are not fully understood, especially in the sensitivity or resistance of target cells to GC. It would be tempting to consider that this Ca^2+^ signaling, induced after stimulation of GC, could be involved in the destruction of cells treated by GC. However, this view would be reductive with respect to Ca^2+^ signaling, a very early event that occurs in the first seconds or even minutes after stimulation of cells with GC. As a second messenger, Ca^2+^ represents a key regulator in survival and cell death. Indeed, we previously reported evidence that chelation of this intracellular Ca^2+^ mobilization would increase the sensitivity of leukemic cells to GC. This suggests its involvement in a cell survival pathway that is therefore resistant to the devastating effect of GC. Furthermore, we and others have provided evidence that Ca^2+^ signaling might induce GC resistance in ALL. In fact, GC-induced Ca^2+^ increases are greater in resistant cells compared to sensitive cells [37,56].An additional pathway by which GC induces its own resistance is through serum GC-inducible kinase-1 (SGK1). In addition to promoting tumor growth, SGK1 signaling contributes to GC resistance [58,59], consistent with its aberrant upregulation found in GC-resistant ALL [60]. Remarkably, SGK1 activates several Ca^2+^ channels and modulators, including Orai1 and Stim [61,62], the main Ca^2+^ pathway involved in lymphocyte activation. SGK1 phosphorylates the protein ubiquitin ligase Nedd4-2, then binds with the protein 14-3-3, and blocks its ability to ubiquinate Orai-1 [62], resulting in increased Orai1 activity, which confers survival of tumor cells [63]. The voltage-sensitive K+ channel, Kv1.3, underlies sustained Ca^2+^ entry via Orai1/STIM1 caused by membrane repolarization [64,65]. Kv1.3 is robustly expressed in ALL [66,67] and upregulated by SGK signaling [68]. Given these observations, a possible hypothesis is that SGK1-mediated activation of Kv1.3 by GC may reflect a mechanism of GC resistance in ALL via a promotion of Ca^2+^ entry [65].

### 4.2. IL-7 Dependent Pathway

IL-7 is a critical actor in the ALL microenvironment. In fact, it promotes the survival and differentiation of thymocytes and the IL-7 receptor/ JAK/STAT5 (IL-7R/JAK/STAT5) signaling pathway contributes to ALL pathogenesis [69,70]. IL-7 positively regulates the expression of the anti-apoptotic gene, BCL2, which promotes thymocyte survival. Conversely, GCs are known to downregulate expression of BCL2 [29,71] leading to pro-apoptotic factors. Recently, At the mechanistic level, the relationship between steroid resistance and IL7R-driven cell survival has recently been proposed in ALL patients [40,72]. Thus, it was shown that GCs elicit their own resistance [39] in the presence of IL-7, by promoting upregulation of IL-7 receptor (IL-7R) expression, resulting in increased STAT5 activation, which consequentially enhances the upregulation of the pro-survival gene BCL-2 [39,40,73,74,75] and the PIM1 kinase gene [76,77]. In addition to the downstream activation of the PI3K-AKT and STAT5 pathways, IL7R signaling also results in the activation of MAPK-ERK signaling [72,78,79,80,81,82]. It has been demonstrated that these signaling pathways can individually contribute to steroid resistance in pediatric ALL via different mechanisms. Activation of STAT5 promotes cell survival through a Bcl-2-independent mechanism [69], instead of activating Bcl-2, STAT5 can activate the PI3K/AKT/mTOR intracellular signaling pathway and leads to leukemia progression [69,70]. As recently suggested, upon GC treatment, strong upregulation of BIM, a pro-apoptotic target gene of GR, may counteract GR/STAT5-induced BCL-XL and BCL2 activation downstream of IL-7-induced or pathogenic IL7R signaling, [40,83]. Consistent with this, recent studies show that JAK-STAT overexpression inhibits GC hypersensitivity by increasing Bcl-2 transcription in ALL cells [84].

### 4.3. PI3K/AKT/mTOR Signaling

The phosphoinositide 3-kinase (PI3K)/Akt pathway plays a huge role in integration of survival signals including cell metabolism, cell cycle progression and proliferation [85]. PI3Ks transduce signals subsequent to growth factors and cytokines; they transform these signals into intracellular messages by generating phosphatidylinositol-3,4,5 trisphosphate (PIP_3_) from phosphatidylinositol-3,4 bisphosphate (PIP_2_) at the membrane. PIP_3_ activates the phosphoinositide-dependent kinase-1 (PDK-1) and the serine–threonine protein kinase (AKT) [86]. Following activation, AKT controls expression of several apoptotic genes. For example, AKT inhibits a pro-apoptotic Bcl-2 family member (BAD)-mediated apoptosis [87,88], prevents XIAP (an anti-apoptotic factor) autoubiquitination and degradation and drives cell survival [87]. AKT activation may result in an increased metabolism which can antagonize the metabolic inhibition induced by GC [89]. mTOR activation by AKT compromises GC-induced apoptosis by increasing the expression of anti-apoptotic MCL1, a Bcl-2 family member [1]. It was demonstrated that FoxO transcription factors can regulate lymphocyte apoptosis and, therefore, play a huge role in GC-induced apoptosis in these cells [48]. BIM is usually induced after GC treatment of cells but an abnormal activation of Akt results in an inhibition of the FoxO3a/Bim pathway leading to downregulation of BIM expression and a defective GC-induced transcription [48]. C-MYC oncogene activation has also been shown to be a signaling pathway associated with GC resistance [90]. 

In ALL, stimulation of tumor cell viability and proliferation, resulting from constitutive activation of the PI3K pathway, has been shown to counteract drug sensitivity in vitro [81,91]. Interestingly, it has been demonstrated that activation of the GR by GC promotes its interaction with PI3K, resulting in TNF-α activation [92], which, by activating the nuclear factor kappa B (NF-KB), promotes ALL cell development and survival [93]. Disruption of GR/PI3K interaction reduces GC effects, suggesting that some functions regulated by GR might occur through kinase interaction [92]. Furthermore, as a determinant of GC resistance, the AKT pathway induces GR phosphorylation (non-functional form) at S134 to prevent its nuclear translocation and result in blockage of transcriptional regulation of GC targets genes [11]. The fact that AKT signaling inhibits GR nuclear translocation following GC treatment indicates that GCs paradoxically activate AKT to regulate GR phosphorylation and prevent GC-mediated cell death [11]. Indeed, functional studies have shown that AKT inhibition facilitates GC-mediated translocation of GR to the nucleus, leading to an absence of GC resistance in vitro and in vivo [11]. Moreover, by activating AKT, GC leads to FoxO3a inhibition [94], activity which is required for GC-induced cell death [95], resulting in its own resistance [48]. In another way, as described above, upregulation of IL-7R pathway by GC [39], in turn, via PI3K stimulation, leads to the activation of Akt/mTOR axes, involved in ALL cell viability, growth, survival and proliferation [80,96,97].

### 4.4. MAPK Signaling Pathway

Studies about crosstalk between the GC and MAPK pathways show a direct role for MAPK for GR signaling [25]. ERK, JNK and p38 are three of the major classes of MAPK [49]. ERK and JNK seems to protect cells against GC-dependent apoptosis. On the other hand, p38 promoted GC-mediated apoptosis. In fact, p38 could phosphorylate GR on serine 211, resulting in enhanced transcriptional and apoptotic activity [49]. Regarding the ERK and JNK pathways, a GC-resistant T cell line, CEM-C1-15, shows high ERK/JNK phosphorylation/activity after GC exposure, whose inhibition confers a Dex-sensitive phenotype [49,50], consistent with the hypothesis that ERK/JNK induce a paradoxical protective effect against Dex-dependent apoptosis [50,98]. Moreover, MEK1 and MEK2 are known to phosphorylate ERK and enhance this activity, leading to cell survival [99]. In T-ALL cells, IL-7 induces activation of the MEK-ERK pathway [80]. Thus, GC, by upregulating IL-7 signaling through IL-7R [44], leads to activation of the MEK/ERK pathway, a cell survival pathway [78,82]. Also, Ca^2+^ signaling is known to phosphorylate ERK [100]. We showed previously that GCs induce their own resistance in B-ALL cells by paradoxically eliciting a Ca^2+^ signaling-mediated pro-survival process that results in activation of ERK pathway. Thus, this activation counteracts the negative action of GCs in B-ALL [37].

### 4.5. Lck Signaling Pathway

Lck is a proto-oncogene, which was found to be overexpressed and hyperactivated in both T-ALL [2] and B-ALL [45,101], where it plays an essential role in cell survival, proliferation and activation [102]. Consistent with this finding, a correlation has been observed between higher basal Lck expression and poor clinical response to GCs in ALL, as measured by phosphoproteomic profiling. This analysis identified that newly diagnosed pediatric ALL patients with a poor response to prednisone tended to have higher Lck expression than did patients with a good response to prednisone [2,103]. Moreover, supporting these observations, Lck hyperactivation has recently been reported in early pediatric T-ALL patients with poor response to initial GC therapy [104]. These findings suggest that LCK-driven oncogenic signaling may be aberrantly activated in ALL, and thus, may play a role in GC resistance. Interestingly, Dex treatment induces Lck phosphorylation at Y394, resulting in a profound activation of several downstream mediators (such as Fyn and ZAP70 kinases) in Jurkat T-ALL cells [44,105]. Maximal Lck phosphorylation occurs between 5 and 15 min after Dex exposure, suggesting a transcription-independent mechanism [44]. Similarly, Dex treatment appears to increase the enzymatic activity of LCK by decreasing its phosphorylation in Y505, resulting in phosphorylation of ZAP70 [44,46].

Other signaling pathways such as IL-7R and *PAX5* activated by GC represent additional LCK-mediated processes that may lead to therapy-induced resistance. Indeed, LCK activation drives IL-7R signaling, a paradoxical resistance pathway activated by GC [39], through STAT5 activation and its downstream target genes cMYC and CCND2. Furthermore, STAT5 signaling is attenuated by LCK inhibition in *PAX5* translocated BCP-ALL patients [101]. *PAX5* alterations have been frequently detected in 30% of ALL patients [45,101], and cases with PAX5 have been found to have a specific driver activity signature and LCK hyperactivation [45]. This is supported by the observation that Dex exposure increases *PAX5* mRNA expression in ALL cells [50]. This activation of LCK signaling by Dex was surprising and paradoxical, considering the established role of Dex as therapy and inductor of cell death in ALL. These findings were confirmed in patients with a good response to prednisone, whose cells showed that TCR engagement-mediated LCK activation was able to limit drug sensitivity. [2]. Furthermore, improvements in GC sensitivity or reversal of GC resistance has been observed following LCK inhibition, both in cell lines and pediatric primary ALL [2,43,45,46,106].

In addition, others studies show that in chronic lymphocytic leukemia (CLL), GC resistance is mediated by LCK, through the aberrant expression of the lymphocyte cell-specific tyrosine kinase (Lck), [107]. Activation of LCK leads to its translocation to the membrane to phosphorylate TCR [108] and BCR. As a result, the phospholipase C signaling pathway is activated and production of IP_3_ results in the release of Ca^2+^ from the endoplasmic reticulum into the cytoplasm through IP_3_ receptors [109]. Lck can also regulate intracellular Ca^2+^ signals by directly activating IP_3_ channels [110]. Following this, calcineurin is activated by IP_3_-mediated Ca^2+^ signaling, leading to translocation and nuclear activation of NF-AT and proinflammatory cytokines such as Il-4 [2,110]. Importantly, calcineurin activation protects cells from GC-induced apoptosis [111]. Lck is also hyperactivated in GC-resistant T-ALL patients which lead to an IL-4 overexpression and resistance to GC [2]. 

### 4.6. Hedgehog Signaling

In addition to regulating tissue homeostasis and early T-cell development, the Hedgehog (Hh) signaling pathway contributes to cell cycles and stem cell biology maintenance during embryogenesis [52]. Few studies in the literature have focused on the role and physiological function of the Hedgehog pathway in ALL. Nevertheless, Hh mutations and active signaling have been reported in T-ALL patients [112,113]. Interestingly, it has been shown that Hh pathway contributes to the T-ALL cell growth and survival [51,52] through BCL-2 activation [114]. Regarding the role of GC in Hh signaling, GC positively regulates Hh pathway activation in ALL cell line CEM and inhibition of this pathway by a PKA inhibitor contributes to increased GC-induced cell death [51]. This hypothesis was confirmed both in T-ALL cell lines and in PDX samples showing that inhibition of GLI, the key transcription factor of Hh signaling, enhanced GC-induced cytotoxicity [52]. This protective effect of Hh signaling in GC-induced cytotoxicity has been also demonstrated in others models [115].

As reviewed by Martelli et al. [116], therapeutically relevant signaling pathways interacting with Hh signaling have been identified in T-ALL cells such as the Notch pathway. This plays an important role in leukemic cell growth, survival, and rewired metabolism in both T-ALL [117,118] and B-ALL cells [119]. Oncogenic *Notch1* mutations are common in T-ALL and can be detected in 50% of cases [118]. *Notch1* oncogenic activity controls a feed-forward loop that promotes cell growth by inducing *c-MYC* gene expression [118,120] and confer primary GC resistance [117]. *Notch1* also regulates other survival signaling pathways such as PI3K-AKT [121], Bcl-2 [117] and IL-7Rα [122] pathways. 

The Hh pathway is active in T-ALL cells and it crosstalks with *Notch* and GC signaling pathways [52]. A combination treatment consisting of a *Notch* inhibitor (γ-secretase inhibitor or GSI) and a Hh inhibitor (cyclopamine) downregulates the active fragment of *Notch* (intracellular NOTC, ICN) expression in suppressing the growth of T-ALL cell lines [123]. Moreover, GANT61, a Hh/GLI inhibitor, has demonstrated high cytotoxicity in *Notch*-dependent T-ALL [124]. Therefore, these studies suggest that crosstalk between the two signaling pathways, Hh and *Notch*, contribute to an additive or synergistic effect on the growth of ALL cells. It cannot be ruled out that part of the effects of GCs on the Hh pathway leading to GC-resistance are related to activation of the *Notch* pathway.

Other signaling pathways (PI3K/Akt/mTOR and MEK/ERK) interact with the Hh pathway. Inhibition of Akt or ERK pathways decreased GLI1 expression levels, suggesting that suggesting a positive regulation of GLI1 transcriptional activity and expression by these two pathways [125], the mechanism of which remains to be explored [116]. However, regarding ERK pathway, it has been shown that ERK2 phosphorylates GLI1 at three sites (Ser102, 116, 130), leading to weakening of GLI1 binding to SUFU, thereby increasing GLI1 transcriptional activity [126]. Thus, the activation of the Hh signaling pathway by GC may be involved in the activation of multiple cell survival signaling pathways.

### 4.7. Metabolic Reprogramming

In different human tissues, endogenous GC play an essential role in the regulation of energy metabolism in physiological or pathological conditions. Transcriptional profiling of GC-resistant leukemia cells revealed additional factors that may contribute to resistance: glutaminolysis, oxidative phosphorylation (OXPHOS) and glycolysis [89]. Considering glycolytic metabolism in cancer cell reprogramming, Olivas-Aguirre et al. recently reported evidence that GCs trigger a pro-survival mechanism in resistant cells. This mechanism involves a metabolic shift from glycolysis and glutaminolysis to increased lipolysis and fatty acid oxidation [56]. Accordingly, inhibition of this metabolic pathway greatly increased ALL cells sensitivity to GC and overcame GC resistance [56].

## 5. Inhibition of Paradoxical Signaling Nodes to Overcome GC Resistance

Overcoming resistance to GCs has become a necessity to improve ALL patients’ outcomes, but there are hurdles in finding effective approaches or treatments. It is increasingly accepted that GR genetic variations are not linked to GC resistance of ALL cells, but rather through deregulated signaling pathways [127,128,129]. These signaling pathways involved in the regulation of proliferation, differentiation, apoptosis, proteostasis, metabolism, autophagy, oncogenes and epigenetic modifications or tumor suppressors represent processes that may lead to therapy-induced resistance. Indeed, many functional studies as described in this report support the hypothesis that resistance to GCs is not only mediated by constitutive activity of these signaling pathways, but that they are also paradoxically activated by the GC themselves, thereby counteracting their therapeutic effect. Thus, it is not surprising that targeting these paradoxical pathways by promising small molecule inhibitors would overcome the resistance and increase the response of ALL cells to GCs as presented in Table 3. We present hereafter evidence for the modulation of these interconnected paradoxical pathways (identified in the previous section) as a mechanism for overcoming GC resistance.

### 5.1. Inhibition of Ca^2+^ and MAPK Signaling

In healthy lymphocytes as well as in ALL cells, Ca^2+^ signaling plays an important role in cell proliferation [53,54,55]; therefore, Ca^2+^-mediated survival can be targeted directly or indirectly to improve GC sensitivity and apoptosis. We have previously shown that GC-induced intracellular Ca^2+^ inhibition or chelation with Bapta-AM significantly potentiates, whereas the increase in cytosolic Ca^2+^ by thapsigargin suppresses the sensitivity of ALL cell lines and primary specimens to GC treatment [37]. We further demonstrated that a specific TRPC3 blocker, Pyr3, leads to a decrease in GC-induced Ca^2+^ signal, thereby promoting GC-mediated cell death [38,130]. Furthermore, Ca^2+^ signal induction is able to activate ERK1/2 protein and decrease GC responsiveness by limiting mitochondrial-mediated apoptotic signals [37]. Therefore, inhibition of MAPK pathways is expected to suppress ALL cell growth. For example, inhibition of a MAPK pathway (ERK1/2, MEK1/2, MEK2 or MEK4) increased ALL cells sensitivity to GC [37,72,99,131]. In addition, MEK overexpression induced ALL cells resistance to GC [131]. Furthermore, inhibition of JNK (another MAPK-signaling pathway), with SP600125 re-sensitizes GC response in ALL [50].

### 5.2. Inhibitor of PI3/AKT/mTOR Pathway

PI3K/AKT/mTOR modulation is expected to overcome resistance in ALL [48,86,132]. Several ALL cell lines and patients with ALL-derived xenograft samples treated with PI3K inhibitor AS605240 revealed a synergetic effect with GC [133]. Further experiments demonstrated that inhibition of AKT can reverse GC resistance by restoring GR translocation to the nucleus [11,132]. Akt2 inhibition has been shown to be more effective in restoring GC sensitivity than Akt1 inhibition, demonstrated by a higher synergistic effect with dexamethasone and less hepatic cytotoxicity [48]. Selective mTOR kinase modulators that target the catalytic subunit of mTor and lead to a pro-apoptotic and anti-proliferative phenotype [134]. As recently developed in ALL preclinical studies, the dual PI3K-mTOR inhibitor BEZ235 controls apoptosis markers [135] such as expression of pro-apoptotic BIM and anti-apoptotic MCL1, thereby restores GC sensitivity [136]. 

### 5.3. Inhibitor of IL-7R and BCL-2 Signaling

**Figure 2:**
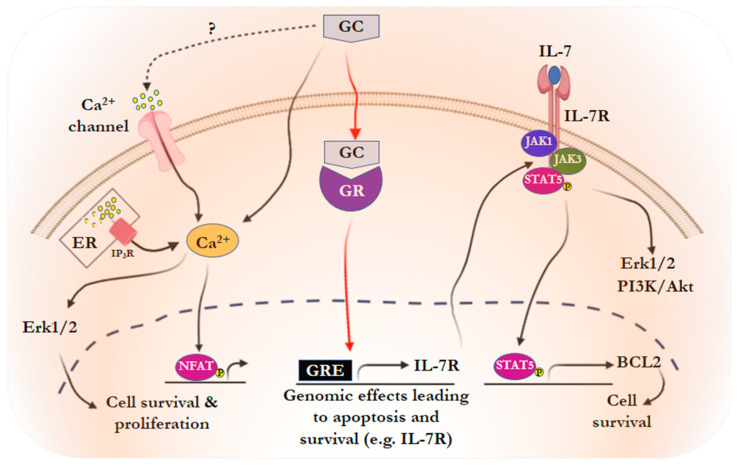
Proposed model for the mechanism by which GC induce their own resistance ALL cells. Dex treatment leads to GR translocation, which induces a transcriptional program resulting in lymphoid cell death (therapeutic effect). Along with this pathway, Dex also provokes cytosolic Ca^2+^ release as well as IL-7R upregulation and subsequent triggering of a pro-survival activity (paradoxical effect). This latter pathway may blunt the anticancer efficacy of chemotherapy (for other details see text).

We previously saw that IL-7-mediated GC resistance involves an increase in pro-survival BCL-2 signaling via the IL-7R/JAK/STAT5 pathway (Figure 2) [39,75]. In this state of mind, inhibiting these actors in this pathway can directly antagonize GC-induced apoptosis. For example, disruption of the Sec61 translocon, which prevents IL-7R from reaching the membrane surface, overcomes GC resistance in ALL [137]. Moreover, JAK1/2 inhibition as well as BCL-2 inhibition can effectively overcome GC resistance in ALL [39,40,69,138,139]. A loss of BCL2 phosphorylation was correlated with JAK3 inhibition with tofacitinib. The synergy between tofacitinib and conventional chemotherapies, including GC, has been suggested as a good strategy for ALL, in vitro and in vivo [42]. Consistently, paraoxonase-2 silencing, which expression was correlated with reduction of Bcl-2 expression, inhibits tumor growth by sensitizing ALL cells to GC in vivo and in vitro [140].

### 5.4. Inhibition of Hedgehog Signaling

Recent report demonstrated that inhibition of Hedgehog signaling via GLI inhibitor GANT61 was shown to exert a synergistic anti-leukemic effect with GC in ALL cell lines and patient-derived xenografts samples [52]. Moreover, inhibition of Hedgehog activity increases ALL cells sensitivity to GC [51]. Likewise, NOTCH1 signaling promotes cell cycle progression and limits GC sensitivity [141]. Thus, inhibition of this pathway, through γ-secretase inhibitors (GSI), reverses GC resistance by upregulation of the GR and the pro-apoptotic BIM proteins [142,143].

### 5.5. Lck Inhibition

Lck inhibition has shown encouraging results. For example, pharmacological inhibitors such as dasatinib, nintedanib, WH-4-023 and bosutinib or specific Lck gene silencing, were shown to induce cell death in GC-resistant ALL cells, and combined treatment with GCs is able to reverse GC resistance in vitro, ex vivo, in vivo and in PDX samples [2,43,45,46,47]. This improvement in GC sensitivity is correlated with an increase in GRs, following inhibition of Lck, which means that Lck exerts a negative action on GR [46]. In addition, inhibition of Lck or aberrantly expressed Lck reverses GC insensitivity in chronic lymphocytic leukemia [107].

## 6. What Direction to Go in to Decipher this Paradox and Move the Field Forward

Although outcomes for children with ALL have dramatically improved over the past decades, some patients are treatment refractory and resistant to GCs. Many studies have focused on understanding the mechanisms of acquiring GC resistance observed during treatment, including genetic or epigenetic alterations in ALL cells [1,144]. Upfront detection of therapy-resistant pathways at the time of diagnosis may allow for treatment adaptation and relapse prevention. Indeed, treatment of ALL by GCs is also limited by primary resistance, i.e., from the upfront GC treatment [26,27]. Therefore, the initial behavior of cells in response to treatment is a major predictor of the efficacy of chemotherapy and the long-term outcomes of patients with B-ALL [145]. The phenomenon of resistance is responsible, in about 20–25% of patients, for relapse [146]. Resistance may justify therapeutic escalation to, for example, hematopoietic stem cell transplantation (HSCT) and/or chimeric antigen receptor (CAR) T cell transplantation. This resistance is observable from the first phase of treatment with GCs as monotherapy [35,36,40], suggesting the existence of intrinsic differences in GC sensitivity in ALL patients at the time of disease diagnosis. These differences can have long-term prognostic significance. This hypothesis is consolidated by the Berlin–Frankfurt–Münster (ALL-BFM) 95 trial, in which ALL patients were stratified into two groups following 7 days of prednisone monotherapy: those who had a prednisone poor response (PPR) and those who had a prednisone good response (PGR). PPR patients had an 8-year event-free survival rate of only 55.1%, as opposed to 81.3% for patients with a PGR [35]. Consequently, understanding the mechanistic basis for intrinsic differential resistance to GC, preventing or reversing it could mean a real breakthrough in the treatment of ALL. Among these mechanisms, attention should be paid to the cell survival pathways paradoxically activated by the GC themselves. Some recent studies have highlighted this paradoxical behavior of GC by the discovery of certain pathways such as Ca^2+^ signaling, IL-7R, PI3/AKT, MAPK/ERK and Hedgehog signaling. Still, further experiments are needed to identify these activated pathways from the upfront GC treatment. Then, it will be necessary to identify all the key molecules of these pathways that are paradoxically activated by the therapy before proceeding to development of a personalized therapy program in which patients whose cells strongly express these targets are treated by the combination of treatment with modulators of the identified pathway and first-line GC. 

## 7. Conclusions

The therapy of ALL has been the subject of considerable efforts over the past decades. Despite this, many children and adult patients relapse. Resistance to corticosteroids is one of the main causes of major failure of the induction phase and relapse. This indicates an urgent need to understand the underlying resistance mechanisms and explore ways to circumvent them. Current efforts have provided valuable knowledge to understand these mechanisms and to develop small molecule inhibitors specifically to improve GC therapy. The best way to improve research on this subject would be to increase the understanding of cell survival pathways paradoxically activated by the GC themselves in order to prevent possible relapses. However, other escape mechanisms remain to be discovered. Focus on rational and feasible combinations of these pathways is essential and necessary to limit toxicity and to improve patients’ quality of life and the general status of patient outcomes.

## Figures and Tables

**Table 1:** GC used in leukemia therapy.

Name	GC Potency	Mineral Corticoid Potency	Plasma Half-life	Dosing in Treatment	References
Prednisone	3.5–4	0.8	12–36 h	40–60 mg/m^2^	[14,15]
Dexamethasone	25–80	0	36–54 h	6–10 mg/m^2^	[14]

**Table 2:** Clinical trials utilizing GC.

Trials	n	GC	Dose	Duration of Treatment	Disease	Study Arm/Group	Results	Refs
NCT00613457	2039	Dex Pred	10 mg/m^2^/day 60 mg/m^2^/day	14 days 20 days	ALL	Pred vs. Dex	Dex reduced the incidence of better salvageable relapses Significant survival benefit from dex only for patients with T-cell ALL	[16]
NCT03390387	4000	Dex Pred	6 mg/m^2^/day 60 mg/m^2^/day	28 days or day 1–15 and 22–29 28 days	ALL	Dex intermittent vs Dex continue vs. Pred	Recruiting	NA
NCT00002816 Phase 3	120	Dex	-	-	Relapsed ALL	Early relapse vs. late relapse for drugs association	After reinduction, LPC counts were lower than in patients treated for an overt BM first relapse.	[17,18]
NCT00707083 Phase 3	2231	Dex	-	Days 1–5 and 29–33	ALL		Bone marrow suppression and liver toxicity	[19]
(CCG)-1922	1060	Dex Pred	6 mg/m^2^/day 40 mg/m^2^/day	28 days 28 days	ALL	6 MP + Oral Pred vs. 6 MP + Intravenous Pred vs. 6 MP + Oral dex vs. 6 MP + Intravenous Dex	Dex provided a 34% reduction in risk of relapse	[20]
AALL0232	3154	Dex Pred	10 mg/m^2^/day 60 mg/m^2^/day	14 days 28 days	High risk B-ALL	Pred vs. Dex	Higher rate of subsequent osteonecrosis with Dex-treated patient	[21]
NCT00003728	1947	Dex Pred	6 mg/m^2^/day 60 mg/m^2^/day	-	ALL	Pred vs. Dex	EVS similar Decreased cumulative incidence of CNS relapse with dex.	[22]
NCT01324180	14	Dex	10 mg/m^2^/day	-	Relapsed/ refractory ALL	Met + VPLD	-	NA
NCT03613428	12	Pred	1 mg/kg	28 days	Relapse/refractory T-ALL	Rux + Pred	-	NA
NCT03817320	31	Dex	10 mg/m^2^/day	14 days	Relapsed /refractory ALL	Ixa + VXLD	Recruiting	NA

n: number of patients; Dex: Dexamethasone; Pred: Prednisone; Met: Metformin; Rux: Ruxolitinib; VLPD: Vincristine, Dexamethasone, Doxorubicin, and PEG-asparaginase; Ixa: Ixazomib; VXLD: Vincristine, Dexamethasone, Asparaginase, and Doxorubicin; EFS: Event free survival; CNS: central nervous system; NA: no publication available; LPC: leukemic progenitor cell.

**Table 3:** GC-activated signaling pathways in ALL whose inhibition overcomes GC resistance or increases GC sensitivity.

Pathways	Drug	Activation Mode	Inhibitor (s)	Leukemic Cells	Consequences	References
Ca^2+^ signaling	Dex	intracellular release	BAPTA-AM Pyr3 thapsigargin	Reh Nalm-6	increases GC sensitivity	[37,38]
IL7R signaling	Dex	IL-7R upregulation	Ruxolitinib JAK3i	CCRF-CEM Patient samples PDX samples	overcomes GC resistance	[39,40]
BCL2 signaling	Dex	BCL2 upregulation	ABT-199 Ruxolitinib Tofacitinib	PDX samples Patient samples	overcomes GC resistance	[39,41,42]
LCK signaling	Dex/Pred	LCK phosphorylation	Dasatinib Bosutinib Nintedanib WH-4-023 shRNA	Patient samples CCRF-CEM Jurkat TALL-1 SUPT1 LK203 PDX samples	overcomes GC resistance	[2,43,44,45,46,47]
AKT signaling	Dex	AKT phosphorylation	MK2206 Akt inhibitor IV	Patient samples CCRF-CEM MOLT3 PF382	increases GC sensitivity	[11,48]
ERK signaling JNK signaling	Dex	ERK, JNK phosphorylation	U0126 SP600125 ip	CEM-C1-15 R3F9	overcomes GC resistance	[49,50]
Hedgehog signaling	Dex	Hh activation via GLI1 and PTCH mRNAs	mPKI GANT61	CEM-C7-14 T-ALL cell lines PDX samples	increases GC sensitivity	[51,52]

Ca^2+^: Calcium; ip: JNK inhibitory peptide; Dex: dexamethasone; Pred: prednisolone; PDX: Patient-derived xenografts.
